# Translation and validation to Portuguese of a 60-item questionnaire to evaluate theoretical knowledge in fundus examination

**DOI:** 10.5935/0004-2749.2022-0015

**Published:** 2022-10-19

**Authors:** Gabriel Ayub, Breno Di Gregorio, Nelson Wolf, Milena Yonamine, José Paulo Cabral de Vasconcelos

**Affiliations:** 1 Department of Ophthalmology, Faculdade de Ciências Médicas, Universidade de Campinas, Campinas, SP, Brazil

**Keywords:** Questionnaire and surveys, Translating, Fundus oculi, Inquéritos e questionários, Tradução, Fundo de olho

## Abstract

**Purpose:**

To translate and validate a questionnaire that evaluates the theoretical knowledge regarding fundus examination.

**Methods:**

A 60-item multiple-choice English questionnaire that investigates various aspects of knowledge regarding fundus examination was translated into Portuguese. The process involved translation, back-translation, and evaluation by an expert committee. The resulting questionnaire was applied to final-year medical students and ophthalmology residents. Each included subject answered the questionnaire twice, with an interval of one week between each application. Internal consistency, test-retest reliability, inter-rater reliability, and percentage agreement were calculated.

**Results:**

Thirty participants were included (25 medical students and 5 ophthalmology residents). The pass-fail cutoff was calculated at 46, the theoretical false positives were 8.7% and the theoretical false negatives were 2.8%. The observed false positive and false negative rates were 0%. Among the 60 items, test-retest reliability was strong in 17 items, which one had a negative correlation, moderate in 14 items, which one had a negative correlation, and weak in 29 items; inter-rater reliability of 34 items was under 0.4, 17 items were between 0.4 and 0.6, and 8 items were above 0.6. One item had a negative kappa. Among the percent agreement, 10 items were between 40%-60% agreement, 50 were above 60% agreement, and 18 were above 80%. Cronbach’s alpha was calculated as 0.674.

**Conclusions:**

The translated questionnaire provided a standard instrument for future research and interventions to improve medical education in ophthalmology.

## Abstract (pt)

**Objetivos:**

Traduzir e validar para o português um questionário para avaliar o conhecimento teórico no exame de fundo de olho.

**Métodos:**

Um questionário de múltipla escolha de 60 questões em inglês, ao qual avalia diversos aspetos do conhecimento no exame de fundo de olho, foi traduzido para o português. O processo envolveu uma tradução, re-tradução e avaliação por um comitê de especialistas. O questionário resultante foi aplicado em estudantes de medicina do último ano e médicos-residentes em oftalmologia. Cada indivíduo respondeu o questionário duas vezes, com um intervalo de uma semana entre cada aplicação. A consistência interna, a confiança teste-reteste, a confiança entre avaliadores e a porcentagem de concordâncias foram calculadas.

**Resultados:**

Trinta participantes foram incluídos (25 estudantes de medicina e 5 residentes em oftalmologia). A nota de corte para aprovação-reprovação foi calculada em 46, sendo os falsos positivos teóricos de 8,7% e os falsos negativos teóricos 2,8%. No mesmo corte, os falsos positivos e falsos negativos observados foi de 0%. Dentre os 60 itens, a confiança teste-reteste foi forte em 17 itens, sendo que um apresentou uma correlação negativa, moderada em 14 itens, sendo que um apresentou uma correlação negativa, e fraca em 29 itens; a confiança inter-examinador foi inferior a 0,4 em 34 itens, entre 0,4 e 0,6 em 17 itens, e acima de 0,6 em 8 itens. Um dos itens apresentou um valor negativo. A porcentagem de concordância foi entre 40-60% em 10 itens, superior a 60% em 50 itens e acima de 80% em 18 itens. O alfa de Crombach foi calculado com 0,674.

**Conclusões:**

O questionário traduzido propicia um instrumento padronizado para futuras pesquisas e intervenções às quais visem aprimorar o ensino em oftalmologia.

## INTRODUCTION

Blindness from reversible causes has been increasing globally. In 2020, 237 million people were estimated to have a moderate or severe visual impairment, while another 38 million were considered blind^(1)^. Among irreversible causes of blindness, like diabetic retinopathy and glaucoma, an early diagnosis may slow down progression since treatment has been instituted to prevent a severe visual impairment. In this context, fundoscopy emerges as a fast, cheap, and effective strategy for tracking down eye pathologies.

Despite the importance of fundus examination, many doctors currently lack knowledge of the technique and/or self-confidence in performing the examination^(2,3,4,5,6)^. The International Council of Ophthalmology^(7,8)^ recommends knowledge of the basic use and handling of fundoscopy equipment and how to examine and identify the normal and pathological structures. In consonance, the Association of University Professors of Ophthalmology^(9)^ highlights the importance of fundoscopy in primary care and its various applications in prevention and health promotion.

Recently, a questionnaire to measure self-confidence in performing fundoscopy was translated and validated into Portuguese^(6)^. However, at the time, there is no available instruments in Portuguese to measure physicians’ and medical students’ knowledge regarding fundus examination. A questionnaire that evaluates several aspects of fundoscopy such as technique and normal and abnormal findings would help to standardize future research and to propose future interventions to improve medical education in ophthalmology. This study aimed to translate and validate a questionnaire into Portuguese to evaluate theoretical knowledge regarding fundus examination.

## METHODS

This study was approved by the Ethics Committee of the University of Campinas and conducted in compliance with the Declaration of Helsinki. All procedures were fully explained and informed consent was obtained from all participants.

### Translation, cross-cultural adaptation, and validation process

The questionnaire developed by Jørgensen et al.^(10)^ which was used as a reference, consists of 60 multiplechoice questions with 3 answers, of which only 1 is the correct answer. This questionnaire investigates the various aspects of knowledge in the fundoscopy examination, such as the details of the correct technique (items 1-6, 12, 13, 21-26, 43, 45, 56-58, 60), anatomical structures, and pathological findings (items 7-11, 14-20, 27-42, 44, 46-55, 59). Among the 60 questions, 18 were considered easy and 42 to be of moderate difficulty level. The translation, cross-cultural adaptation, and validation process were followed as described in the literature^(11,12,13)^. The reference questionnaire was translated from English to Portuguese by two independent translators, both with high proficiency in English and one with an experience in the fundus examination and ophthalmology. Both the translations were then synthesized and evaluated by an expert committee, which evaluated the equivalence of the translation and made the necessary modifications. The back-translation from Portuguese to English was made by two independent translators with high proficiency in the English language. Both back translations were then compared to the original version of the questionnaire to evaluate the consistency of the translation process. Finally, the expert committee evaluated the final version of the questionnaire (available as Supplemental Material) to verify the semantic, idiomatic, and conceptual equivalence relative to the original questionnaire.

### Test of the translated questionnaire

The resulting questionnaire was applied to final-year medical students and ophthalmology residents. Each subject answered the questionnaire twice, at an interval of 1 week between each application (test-retest). The participants who did not complete the questionnaire at both times were excluded. Internal consistency, test-retest reliability, inter-rater reliability, and percentage agreement were also calculated.

### Statistical analysis

The statistical analysis was performed with the Statistical Package for Social Sciences-SPSS (IBM Corporation, Armon NY, USA, version 22.0). The normality of the mean score of the groups was calculated by Shapiro-Wilk test. The comparison was performed with Mann-Whitney U-test. P<0.05 was considered to indicate statistical significance.

The pass-fail cutoff was calculated with contrasting group methods as described by Jørgensen et al.^(14)^. Internal consistency, to evaluate the inter-correlation of the questionnaire items, was calculated by using coefficient alpha (Cronbach’s alpha-α), which varied from 0 to 1, while a value of >0.7 was considered adequate^(15)^.

Test-retest reliability was calculated by Pearson’s correlation (R), and inter-rater reliability was calculated by Cohen’s Kappa (κ), which varied from 0 to 1, and was classified as <0.2, poor agreement; 0.2-0.4, week agreement, 0.4-0.6, moderate agreement; 0.6-0.8, good agreement; >0.8 excellent agreement^(12,15)^. Percentage agreement was calculated by the number of agreements divided by the total number of answers^(16)^.

## RESULTS

A total of 30 participants were included in the validation process, of which 25 were medical students and 5 were ophthalmology residents. The baseline characteristics are presented in table 1.

**Table 1:** Baseline characteristics of the study subjects.

	n
Instruction level (Medical student/Medical resident)	25/5
Gender (male/female)	16/14
Age (years)	25.5 ± 1.43
Score	39.83 ± 8.11

**Figure 1:**
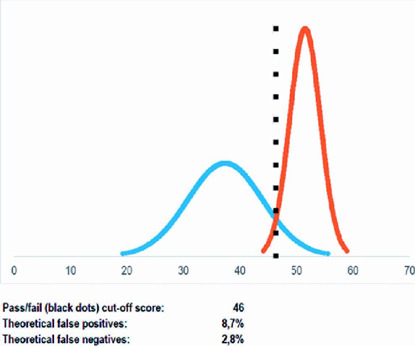
Pass-fail (cutof) score and theoretical false positives and false negatives.

While the overall mean score was 39.83 ± 8.11 (66.38%), medical students’ mean score was 37.48 ± 6.6 (62.46%) and ophthalmology residents’ mean score was 51.6 ± 2.7 (86%) (p<0.0001). The pass-fail cutoff was calculated as 46, the theoretical false positives were 8.7%, and the theoretical false negatives were 2.8% (Figure 1). At this score, the observed rates of false positive and false negative were 0%.

Among the 60 items, test-retest reliability was strong in 17 items having negative correlation, moderate in 14 items having negative correlation and weak in 29 items. The inter-rater reliability was <0.4 in 34 items, between 0.4 and 0.6 in 17 items, and >0.6 in 8 items. Only one item had negative inter-rater reliability. Among the percent agreement, 10 items were between 40%-60% agreement, 50 items were above 60% agreement, and 18 items were above 80% agreement. Cronbach’s alpha was calculated as 0.674. The details of each item are presented in table 2.

**Table 2:** Test-retest reliability (Pearson correlation - R), inter-rater reliability (kappa correlation - κ), and percentage agreement of each question that evaluated the technique (^†^), anatomical, and pathological findings (*)

	R	p-value	κ	p-value	Percentage agreement
Question 1^†^	0.447	0.013	0.444	0.014	83.3
Question 2^†^	0.741	<0.001	0.534	<0.001	69.9
Question 3^†^	0.444	0.014	0.541	<0.001	70.0
Question 4^†^	0.709	<0.001	0.706	<0.001	90.0
Question 5^†^	0.484	0.007	0.380	0.008	83.3
Question 6^†^	0.050	0.795	-0.023	0.786	90.0
Question 7*	0.565	0.001	0.362	0.009	90.0
Question 8*	0.237	0.207	0.282	0.055	70.0
Question 9*	0.209	0.268	0.254	0.104	66.6
Question 10*	0.453	0.012	0.615	<0.001	90.0
Question 11*	0.633	<0.001	0.546	<0.001	70.0
Question 12^†^	0.240	0.202	0.222	0.110	53.3
Question 13^†^	0.247	0.189	0.224	0.083	50.0
Question 14*	0.464	0.010	0.464	0.011	93.3
Question 15*	0.357	0.053	0.268	0.048	70.0
Question 16*	0.526	0.003	0.316	0.017	73.3
Question 17*	0.343	0.064	0.255	0.084	76.6
Question 18*	0.113	0.554	0.286	0.035	66.7
Question 19*	0.747	<0.001	0.652	<0.001	86.7
Question 20*	0.827	<0.001	0.716	<0.001	86.7
Question 21^†^	0.335	0.070	0.389	0.002	63.3
Question 22^†^	0.746	<0.001	0.489	<0.001	70.0
Question 23^†^	0.188	0.321	0.114	0.455	76.6
Question 24^†^	0.233	0.216	0.375	0.018	73.3
Question 25^†^	0.330	0.075	0.330	0.070	66.7
Question 26^†^	0.391	0.033	0.331	0.007	79.9
Question 27*	0.083	0.661	0.180	0.272	60.0
Question 28*	0.778	<0.001	0.471	<0.001	66.7
Question 29*	0.354	0.055	0.286	0.031	86.7
Question 30*	0.447	0.013	0.444	0.014	83.3
Question 31*	0.172	0.362	0.006	0.971	63.3
Question 32*	0.304	0.102	0.688	<0.001	93.4
Question 33*	-0.453	0.012	0.143	0.350	86.7
Question 34*	0.777	<0.001	0.620	<0.001	90.0
Question 35*	0.250	0.183	0.353	0.005	56.6
Question 36*	0.130	0.495	0.106	0.445	46.7
Question 37*	0.901	<0.001	0.773	<0.001	46.7
Question 38*	0.183	0.334	0.318	0.018	60.0
Question 39*	0.500	0.005	0.451	0.001	66.7
Question 40*	0.649	<0.001	0.478	<0.001	76.7
Question 41*	-0.695	<0.001	0.318	<0.001	93.3
Question 42*	0.569	0.001	0.521	0.002	76.7
Question 43^†^	0.859	<0.001	0.817	<0.001	90.0
Question 44*	0.235	0.212	0.339	0.010	66.7
Question 45^†^	0.609	<0.001	0.536	<0.001	70.0
Question 46*	0.255	0.174	0.221	0.103	60.0
Question 47*	0.566	0.001	0.461	<0.001	63.3
Question 48*	0.319	0.086	0.375	0.005	66.7
Question 49*	0.727	<0.001	0.535	<0.001	73.3
Question 50*	0.466	0.009	0.288	0.084	76.7
Question 51*	0.613	<0.001	0.375	0.006	73.3
Question 52*	0.706	<0.001	0.535	0.001	86.7
Question 53*	0.328	0.076	0.137	0.323	60.0
Question 54*	0.044	0.817	0.161	0.255	60.0
Question 55*	0.355	0.054	0.301	0.082	66.7
Question 56^†^	0.324	0.081	0.444	0.009	73.3
Question 57^†^	0.627	<0.001	0.559	<0.001	76.7
Question 58^†^	0.495	0.005	0.339	0.009	56.7
Question 59*	0.270	0.149	0.372	0.010	83.3
Question 60^†^	0.027	0.889	0.083	0.550	66.7

## DISCUSSION

The current study successfully translated a questionnaire into Portuguese that comprehensively evaluated the theoretical knowledge regarding fundus examination. The questionnaire developed by Jørgensen et al.^(10)^ recruited 30 participants for the validation process (20 medical students and 10 ophthalmology specialists). The mean score of the first group was 30.0 ± 4.3 and the mean score of the second group was 57.4 ± 1.6 (p<0.0001). The pass-fail cutoff was calculated as 49.7, which represented the point that no medical student passed the test (no false positive) and no ophthalmology resident failed the test (no false negative). Internal consistency was calculated as 0.95. The validation process revealed that there was a significant difference in the scores between the two groups. A lower pass-fail cutoff (46 vs 49.7) and a lower internal consistency (0.674 vs 0.95) was found in the current study.

Further, reliability is a method to estimate the random errors in an assessment. One way to evaluate reliability is through internal consistency, represented by Cronbach’s alpha. The level of accepted reliability for an instrument depends on its application. For instruments that assess subjects with major consequences for the society such as obtaining a medical license, a value >0.9 is desired; for assessments with moderate consequences such as end-of-year or end-of-course examinations, a minimum of 0.8 is acceptable, and a value <0.7 is acceptable when the instrument aims to assess classroom proficiency^(17)^. In the current study, the reliability was 0.674, close to the minimum 0.7 acceptable for the aim of the questionnaire, which was to evaluate theoretical proficiency in one specific item of the physical examination.

Another method to verify the validity of training and instruments is the proposed contrasting groups’ method^(14)^ which consists of a comparison between two groups with significantly different expertise in the subject, a group of novices and a group of experts. The calculation of the cutoff point and the theoretical false positives and false negatives englobes the mean and standard deviation of the score of each group, which are considered as a normal distribution. This reduces the main issue in validation studies, which is the small sample size, mainly due to the reduced availability of experts. The cutoff point is considered the intersection between the curves of both groups, as shown in figure 1, whereas the theoretical false positive rate is calculated based on the novices who scored higher than the cutoff point, and the false negative rate as the experts who scored lower than the pass-fail point^(14)^. The differences between observed and theoretical false positives and false negatives are due to the assumption of the normal distribution of the values, which may be considered a limitation of the study. The small samples are not always normally distributed, and so the outliers may have a strong influence on the calculations^(14)^. In our casuistic, low rates of theoretical false positives and false negatives were observed, which reflected a good representation of novices with low proficiency, and experts with high proficiency. Both Cronbach’s alpha and contrasting groups are valid methods to calculate reliability^(17)^.

Despite the successful translation and cross-cultural adaptation to Portuguese, the validation process had a few issues. One method of measuring inter-rater reliability was percentage agreement, which was calculated by the number of agreements divided by the total number of answers^(16)^. However, this method did not take into account the chance of agreement, which is a random agreement caused by guessing that resulted in a false agreement. Cohen’s kappa^(18,19)^ was introduced to calculate the agreement including this chance, which varied between -1 and +1, and a value closer to +1 represented a perfect agreement while a value closer to 0 represented no agreement. A negative value represented a disagreement between the observers. Kappa’s correlation also had a few limitations. Despite the minimally accepted values of 0.40-0.59, it represented only a 15%-35% reliability of the data^(16)^, which may be critical values depending on the subject. In our study, we obtained a <0.4 correlation in 35 items and a negative correlation in one item, which represented a limitation of the validation process. However, among the remaining 24 items, a moderate to strong kappa was calculated, which indicated a good agreement and a reliable validation process.

The current study had a few limitations. The population included in the validation process was composed mainly of medical students with moderate to low levels of proficiency in fundoscopy^(2,3,4,5,6)^ which could explain the lower level of agreement by a higher chance of guessing. Also, the extension of the questionnaire composed of 60-items would have influenced the concordance of the answers with a higher rate of concordance in the initial questions and a lower level in the following questions. A shorter questionnaire or a shorter version of the same instrument evaluating the same points would have improved the validation process.

In conclusion, the experts translated and validated the English questionnaire into Portuguese that comprehensively evaluated the knowledge regarding fundoscopy among medical students and ophthalmology residents. Further research is needed to measure the theoretical proficiency regarding fundus examination, which would help to propose effective interventions to improve medical education in ophthalmology.

## Questão 1.

A oftalmoscopia direta é, particularmente, relevante em doenças localizadas:

a. Ao redor do olho

b. Na parte anterior do olho

**c. Na parte posterior do olho**

**Questão 2.**

A distância correta entre o olho do examinador e o oftalmoscópio é importante durante a oftalmoscopia. Essa distância deve ser de:

**a. < 5cm**

b. 5-10cm

c. 10-15cm

**Questão 3.**

Qual é a sequência correta no exame do polo posterior do olho?

a. Encontrar a mácula, depois, os grandes ramos vasculares e, por último, o nervo óptico

b. Encontrar os grandes ramos vasculares, depois, a mácula, e, por último, o nervo óptico

**c. Encontrar o nervo óptico, depois, os grandes ramos vasculares, e, por último, a mácula.**

**Questão 4.**

Uma condição necessária para visualizar a retina é:

a. Um paciente em pé

**b. Estruturas do olho transparentes**

c. Remoção das lentes de contato

**Questão 5.**

Ao examinar o nervo óptico, o paciente é instruído a:

a. Olhar para a luz no oftalmoscópio

**b. Focar em um ponto à sua frente**

c. Olhar para baixo

**Questão 6.**

A abordagem típica para identificar o nervo ótico é:

a. Olhar de cima a baixo

**b. Seguir os vasos sanguíneos**

c. Ajustar a refração

**Questão 7.**

O nervo ótico pode estar edemaciado, condição conhecida como papiledema. Esse fenômeno é caracterizado na seguinte situação:

**a. Borramento na delineação do nervo ótico**

b. Nervo ótico mais brilhante

c. O nervo ótico circundado por pequenos sangramentos

**Questão 8.**

Qual das causas a seguir pode levar ao papiledema?

**a. Hipertesão mal controlada**

b. Aumento da pressão intraocular

c. Infecção nos seios paranasais

**Questão 9.**

Qual das causas a seguir pode levar ao papiledema?

a. Conjuntivite

b. Arritmias cardíacas mal controladas

**c. Isquemia de nervo óptico**

**Questão 10.**

A fóvea é localizada no centro da mácula. Essa área é caracterizada por:

a. Presença de poucos vasos sanguíneos grandes

b. Presença de muitos vasos sanguíneos pequenos

**c. Ausência de vasos sanguíneos**

**Questão 11.**

Qual item a seguir pode ser encontrado em pacientes com arterite temporal?

**a. Papiledema**

b. Relação escavação/disco diminuída

c. Proliferação vascular no nervo óptico

**Questão 12.**

Um paciente é hipermétrope (grau para perto) e usa óculos com +2 de correção. O examinador é míope (grau para longe) e usa óculos com -4 de correção. Nenhum deles tem astigmatismo. Nem o investigador e nem o paciente estão usando óculos durante o exame. Qual a refração a ser selecionada no oftalmoscópio é, teoricamente, ideal durante o exame da retina?

**a. -2**

b. 0

c. +2

**Questão 13:**

Um paciente é hipermétrope (grau para perto) e usa óculos com +2 de correção. O examinador é míope (grau para longe) e usa óculos com -4 de correção. Nenhum deles tem astigmatismo. O paciente não está usando óculos durante o exame. O examinador está usando suas lentes de contato normais. Qual a refração a ser selecionada no oftalmoscópio é, teoricamente, ideal durante o exame da retina?

a. -2

b. 0

**c. +2**

**Questão 14.**

Qual das condições a seguir pode diminuir a visibilidade durante a realização da oftalmoscopia direta?

a. Glaucoma

**b. Catarata**

c. Ambliopia

**Questão 15.**

Qual das seguintes condições pode diminuir a visibilidade durante a realização da oftalmoscopia direta?

a. Conjuntivite

b. Blefarite

**c. Uveíte**

**Questão 16.**

Qual das seguintes condições pode diminuir a visibilidade durante a realização da oftalmoscopia direta?

**a. Sangramento intraocular**

b. Aumento da pressão intraocular

c. Lente artificial de cirurgia intraocular

**Questão 17.**

Qual das seguintes condições pode diminuir a visibilidade durante a realização da oftalmoscopia direta?

a. Oclusão arterial central da retina

**b. Inflamação intraocular**

c. Oclusão venosa central da retina

**Questão 18.**

Qual dos itens a seguir é correto sobre o glaucoma de ângulo aberto?

a. O glaucoma de ângulo aberto pode ser excluído após oftalmoscopia direta

**b. A oftalmoscopia direta não é um exame ideal para detecção do glaucoma de ângulo aberto**

c. O glaucoma de Ângulo aberto pode ser diagnosticado unicamente com a oftalmoscopia direta

**Questão 19.**

Hipertensão arterial grave pode causar mudanças visíveis na oftalmoscopia direta como:

a. Áreas atróficas

**b. Papiledema**

c. Descolamento de retina

**Questão 20.**

Qual dos seguintes tumores primários pode ser visto no polo posterior do olho:

**a. Melanoma maligno**

b. Adenocarcinoma

c. Carcinoma espinocelular

**Questão 21.**

Em qual das mãos o examinador deve segurar o oftalmoscópio ao avaliar o olho esquerdo do paciente sentado?

a. Não há diferença

**b. Mão esquerda**

c. Mão direita

**Questão 22.**

Em qual das mãos o examinador deve segurar o oftalmoscópio ao avaliar o olho direito do paciente sentado?

a. Não há diferença

b. Mão esquerda

**c. Mão direita**

**Questão 23.**

Quando realizado oftalmoscopia direta, na distância correta do paciente, e a retina é visível, porém a imagem não é nítida, a visibilidade pode ser otimizada por:

a. Solicitar que o paciente pisque os olhos por algum tempo

**b. Aumentar ou diminuir a refração definida no oftalmoscópio**

c. Mudar para o modo luz vermelha no oftalmoscópio

**Questão 24.**

Depois da dilatação pupilar, há um risco que um paciente susceptível possa desenvolver:

a. Hipermetropia transitória

**b. Aumento da pressão intraocular**

c. Isquemia da córnea

**Questão 25.**

Há aumento do risco de complicações da dilatação pupilar em:

**a. Pacientes com hipermetropia elevada**

b. Pacientes operados recentemente para catarata

c. Paciente em idade produtiva

**Questão 26.**

É necessário cuidado ao utilizar colírio para dilatação pupilar em paciente com:

**a. Glaucoma de angulo fechado**

b. Opacificação do cristalino

c. Diversas “moscas volantes” no campo visual

**Questão 27.**

Qual é a posição anatômica do nervo óptico no polo posterior do olho em relação ao eixo óptico?

a. Diretamente na linha mediana vertical

b. Lado temporal da linha mediana vertical

**c. Lado nasal da linha mediana vertical**

**Questão 28.**

Escavação nas bordas do disco óptico pode ser visto em pacientes com:

**a. Glaucoma**

b. Degeneração macular relacionada à idade

c. Hipertensão arterial sistêmica

**Questão 29.**

Qual das seguintes causas podem levar ao papiledema?

a. Coriorretinopatia serosa central

**b. Aumento da pressão intracraniana**

c. Iridociclite

**Questão 30.**

Qual a melhor conduta quando detectado papiledema fora de um serviço especializado?

a. Manter seguimento não especializado, com consultas frequentes para avaliar progressão

b. Referenciar ao especialista de rotina

**c. Referenciar ao serviço de urgência**

**Questão 31.**

Qual a melhor conduta quando detectado extenso sangramento intraocular fora de um serviço especializado?

a. Manter seguimento não especializado, com consultas frequentes para avaliar progressão

b. Referenciar ao especialista de rotina

**c. Referenciar ao serviço de urgência**

**Questão 32.**

Pacientes diabéticos podem desenvolver retinopatia diabética. Qual dos seguintes sinais são frequentemente encontrados?

a. Papiledema

**b. Pequenas hemorragias retinianas**

c. Áreas atróficas

**Questão 33.**

Pacientes diabéticos podem desenvolver retinopatia diabética. Qual dos seguintes sinais são frequentemente encontrados?

a. Aumento da relação escavação/disco

**b. Microaneurismas**

c. Oclusão arterial central da retina

**Questão 34.**

Pacientes diabéticos podem desenvolver retinopatia diabética. Qual dos seguintes sinais são frequentemente encontrados?

a. **Lesões brancas algodonosas na retina**

b. Lesões escuras delimitadas na retina

c. Lesões escuras algodonosas na retina

**Questão 35.**

Múltiplos sangramentos seguindo a linha de orientação nervosa da retina podem ser vistos em pacientes com:

a. Aumento importante da pressão intraocular

b. Oclusão arterial central da retina

**c. Oclusão venosa central da retina**

**Questão 36.**

Múltiplos sangramentos seguindo a linha de orientação nervosa em uma das metades da retina podem ser vistos em pacientes com:

a. Aumento discreto da pressão intraocular

b. Oclusão do ramo arterial

**c. Oclusão do ramo venoso**

**Questão 37.**

Qual a melhor conduta quando se suspeita de um descolamento de retina fora de um serviço especializado?

a. Manter seguimento não especializado, com consultas frequentes para avaliar progressão

b. Referenciar ao especialista de rotina

**c. Referenciar ao serviço de urgência**

**Questão 38.**

Qual dos seguintes sinais pode ser encontrado na arterite temporal:

a. Proliferação vascular da retina

**b. Oclusão arterial central**

c. Degeneração macular relacionado a idade

**Questão 39.**

Em pacientes críticos, a oftalmoscopia direta é importante ao avaliar:

a. Circulação: aferição da pressão sanguínea, pulso e ritmo cardíaco

b. Incapacidade: avaliação do estado mental, nervosa e da glicemia

**c. A oftalmoscopia direta não é relevante para avaliação de pacientes críticos**

**Questão 40.**

Antes de se realizar a oftalmoscopia direta, a pupila pode ser visualizada através do oftalmoscópio e pode se observar o reflexo vermelho. A ausência do reflexo vermelho na criança pode ser causado por:

a. Má colaboração ao exame

b. Ambliopia

**c. Tumor intraocular**

**Questão 41.**

Antes de realizar a oftalmoscopia direta, a pupila do paciente pode ser visualizada através do oftalmoscópio, sendo possível observar o reflexo vermelho. A ausência desse reflexo em uma criança pode ser causada por:

a. Descolamento de vítreo

**b. Catarata congênita**

c. Não desenvolvimento da retina

**Questão 42.**

Antes de realizar a oftalmoscopia direta, a pupila do paciente pode ser visualizada através do oftalmoscópio, sendo possível observar o reflexo vermelho. Qual a conduta correta quando não se observa o reflexo vermelho em uma criança fora de um serviço especializado?

a. Manter seguimento não especializado, com consultas frequentes para avaliar progressão

b. Referenciar ao especialista na rotina

**c. Referenciar ao serviço urgência**

**Questão 43.**

Durante o exame da mácula, pode ser uma boa estratégia solicitar que o paciente:

**a. Olhe para a luz no oftalmoscópio**

b. Foque em um ponto à sua frente

c. Olhe para baixo

**Questão 44.**

Uma importante palidez na retina de um olho com um ponto vermelho central (mácula em cereja) pode ser vista em pacientes com:

a. Pressão intraocular bastante elevada

**b. Oclusão de artéria central da retina**

c. Oclusão da veia central da retina

**Questão 45.**

Quanto tempo demora para os colírios dilatadores agirem?

a. 5 a 10 minutos

b. 1 a 5 minutos

**c. 15 a 20 minutos Questão 46.**

Como é calculada a relação escavação/disco?

a. A área do disco dividida pela área da escavação

**b. O diâmetro vertical da escavação em relação ao diâmetro do disco**

c. A área do disco dividido pelo raio horizontal da escavação

**Questão 47.**

O papiledema pode ter aparência similar a outras condições. Entre elas, temos:

a. Degeneração macular relacionada a idade

b. Retinopatia diabética

**c. Fibras nervosas mielinizadas**

**Questão 48.**

O papiledema pode ter aparência similar a outras condições. Entre elas, temos:

**a. Drusas de disco óptico**

b. Glaucoma agudo

c. Microaneurismas

**Questão 49.**

Qual das assertivas está correta em relação aos vasos retinianos?

a. Arteríolas e vênulas retinianas aparentam ter a mesma espessura

b. Arteríolas retinianas aparentam ser mais espessas que as vênulas

**c. Vênulas retinianas aparentam ser mais espessas que as arteríolas**

**Questão 50.**

A artéria central entra na retina através:

a. Da córnea

b. Da mácula

**c. Do nervo óptico Questão 51.**

As vênulas saem da retina através:

a. Da córnea

b. Da mácula

**c. Do nervo óptico**

**Questão 52.**

A fóvea está localizada no centro da mácula. Qual sua função primária?

**a. Visão de alta resolução**

b. Visão noturna

c. Visão essencial para a orientação

**Questão 53.**

Diversos pontos brilhantes podem ser vistos na retina de um paciente com degeneração macular relacionada à idade. Qual a conduta correta quando se suspeita dessa situação em um paciente fora de um serviço especializado:

a. Manter seguimento não especializado, com consultas frequentes para avaliar progressão

**b. Referenciar ao especialista na rotina**

c. Referenciar ao serviço de urgência

**Questão 54.**

Hipertensão arterial severa pode causar alterações observáveis na oftalmoscopia direta. Entre elas, temos:

**a. Sangramentos retinianos próximos ao disco óptico**

b. Proliferação de vasos sanguíneos

c. Palidez de nervo óptico

**Questão 55.**

O manejo correto de um paciente recém diagnosticado com diabetes tipo 2:

a. Realização de oftalmoscopia direta anual por um não especialista

**b. Referenciar ao especialista na rotina**

c. Referenciar ao serviço de urgência

**Questão 56.**

Quantas gotas de colírio dilatador são necessárias em cada olho para observar seu efeito?

**a. 1-2**

b. 3-4

c. 5-6

**Questão 57.**

Descreva a imagem observada na oftalmoscopia direta:

**a. Direita, não invertida**

b. Pronada, de ponta cabeça

c. Virtual, espelhada

**Questão 58.**

Uma boa postura das mãos do examinador inclui:

**a. O dedo indicador no anel de foco e uma superfície de contato com a face do paciente**

b. O uso do descanso do polegar e o dedo do meio no ajuste de luz

c. Uma pegada firme, não cobrindo o indicador de foco

**Questão 59.**

Quais são bons parâmetros para avaliar o disco óptico?

**a. Coloração, margens e escavação**

b. Tamanho, seu conteúdo de mielina e a contagem de células

c. Razão vaso-disco

**Questão 60.**

O que você considera ser um problema relevante da oftalmoscopia direta?

**a. Com o pequeno campo visual, é fácil “se perder” ao explorar a fundoscopia e menosprezar ou cometer equívocos na avaliação do tamanho das lesões**

b. O equador pode ser visto (mais anteriormente), se os raios de luz entram no olho por um ângulo oblíquo

c. O reflexo corneano pode ser movido para fora da imagem retiniana com a mudança do ângulo de visão

## References

[1] Flaxman SR, Bourne RRA, Resnikoff S, Ackland P, Braithwaite T, Cicinelli MV, Das A, Jonas JB, Keeffe J, Kempen JH, Leasher J, Limburg H, Naidoo K, Pesudovs K, Silvester A, Stevens GA, Tahhan N, Wong TY, Taylor HR, Vision Loss Expert Group of the Global Burden of Disease
Study (2017). Global causes of blindness and distance vision impairment
1990-2020: a systematic review and meta-analysis. Lancet Glob Health.

[2] Wu EH, Fagan MJ, Reinert SE, Diaz JA (2007). Self-confidence in and perceived utility of the physical
examination: A comparison of medical students, residents, and faculty
internists. J Gen Intern Med.

[3] Cordeiro MF, Jolly BC, Dacre JE (1993). The effect of formal instruction in ophthalmoscopy on medical
student performance. Med Teach.

[4] Kelly LP, Garza PS, Bruce BB, Graubart EB, Newman NJ (2013). Teaching Ophthalmoscopy to Medical Students (the TOTeMS
Study). Am J Ophthalmol.

[5] Mackay DD, Garza PS, Bruce BB, Bidot S, Graubart EB, Newman NJ (2014). Teaching ophthalmoscopy to medical students (TOTeMS) II: A
one-year retention study. Am J Ophthalmol.

[6] Ayub G, Souza RB, de Albuquerque AM, de Vasconcellos JP (2021). Comparison of conventional and wide field direct ophthalmoscopy
on medical students’ self-confidence for fundus examination: a 1-year
follow-up. BMC Med Educ.

[7] Straatsma BR, Coscas GJ, Naumann GO, Spivey BE, Taylor HR (2001). International ophthalmology strategic plan to preserve and
restore vision-vision for the future. Am J Ophthalmol.

[8] International Task Force on Opthalmic Education of Medical
Students, International Council of Opthalmology (2006). Principles and guidelines of a curriculum for ophthalmic
education of medical students. Klin Monbl Augenheilkd.

[9] Stern GA (1995). Teaching Ophthalmology to Primary Care Physicians. Arch Ophthalmol.

[10] Jørgensen M, Savran MM, Christakopoulos C, Bek T, Grauslund J, Toft PB (2019). Development and validation of a multiple-choice
questionnaire-based theoretical test in direct
ophthalmoscopy. Acta Ophthalmol.

[11] Beaton DE, Bombardier C, Guillemin F, Ferraz MB (2000). Guidelines for the process of cross-cultural adaptation of
self-report measures. Spine (Phila Pa 1976).

[12] Gjersing L, Caplehorn JR, Clausen T (2010). Cross-cultural adaptation of research instruments: Language,
setting, time and statistical considerations. BMC Med Res Methodol.

[13] Sousa VD, Rojjanasrirat W (2011). Translation, adaptation and validation of instruments or scales
for use in cross-cultural health care research: A clear and user-friendly
guideline. J Eval Clin Pract.

[14] Jørgensen M, Konge L, Subhi Y (2018). Contrasting groups’ standard setting for consequences analysis in
validity studies: reporting considerations. Adv Simul.

[15] Tsang S, Royse CF, Terkawi AS (2017). Guidelines for developing, translating, and validating a
questionnaire in perioperative and pain medicine. Saudi J Anesth.

[16] McHugh ML (2012). Interrater reliability: the kappa statistic. Biochem Medica.

[17] Downing SM (2004). Reliability: On the reproducibility of assessment
data. Med Educ.

[18] Cohen J (1960). A Coefficient of agreement for nominal scales. Educ Psychol Meas.

[19] McHugh ML (2012). Interrater reliability: the kappa statistic. Biochem Medica.

